# Bacterial Infection and Immune Responses in *Lutzomyia longipalpis* Sand Fly Larvae Midgut

**DOI:** 10.1371/journal.pntd.0003923

**Published:** 2015-07-08

**Authors:** Matthew Heerman, Ju-Lin Weng, Ivy Hurwitz, Ravi Durvasula, Marcelo Ramalho-Ortigao

**Affiliations:** 1 Department of Entomology, Kansas State University, Manhattan, Kansas, United States of America; 2 Department of Internal Medicine, University of New Mexico School of Medicine Albuquerque, New Mexico, United States of America; 3 New Mexico VA Health Care System, Albuquerque, New Mexico, United States of America; Liverpool School of Tropical Medicine, UNITED KINGDOM

## Abstract

The midgut microbial community in insect vectors of disease is crucial for an effective immune response against infection with various human and animal pathogens. Depending on the aspects of their development, insects can acquire microbes present in soil, water, and plants. Sand flies are major vectors of leishmaniasis, and shown to harbor a wide variety of Gram-negative and Gram-positive bacteria. Sand fly larval stages acquire microorganisms from the soil, and the abundance and distribution of these microorganisms may vary depending on the sand fly species or the breeding site. Here, we assess the distribution of two bacteria commonly found within the gut of sand flies, *Pantoea agglomerans* and *Bacillus subtilis*. We demonstrate that these bacteria are able to differentially infect the larval digestive tract, and regulate the immune response in sand fly larvae. Moreover, bacterial distribution, and likely the ability to colonize the gut, is driven, at least in part, by a gradient of pH present in the gut.

## Author Summary

Symbiotic microorganisms influence many aspects of the physiology of their hosts. In insects, symbiotic bacteria are able among other things to modulate the immune response and the development of the insect from larval stages to adult. Many bacteria first gain access to insect tissues, such as the gut, during larval development, and are acquired from the environment. Thus, depending on the insect ecology, aquatic vs. terrestrial, the bacterial gut flora found in insects can vary widely. Little is known about the events that follow bacterial infection in larval guts and the driving forces for colonization of the gut by such bacteria. We investigated the distribution of two bacteria, a Gram-positive (*Bacillus subtilis*) and a Gram-negative (*Pantoea agglomerans*) fed to sand fly larvae. Our results indicate that bacteria distribution in the larval gut is driven by their ability to multiply at a given pH, as pH in the gut also varies. Gut distribution by these bacteria lead to an immune response that the sand fly larva is able to modulate according to the bacterial species. Our findings can influence development of paratransgenic approaches that utilize bacterial symbionts to control vector population.

## Introduction

Bacterial symbionts significantly influence many aspects of the physiology of their host. In insects, both pathogenic and non-pathogenic bacteria have been shown to modulate immune response, homeostasis, development, and overall health of midgut physiology for both larval and adult stages. Both Gram-positive (G+) and Gram-negative (G-) bacteria are commonly associated with the midgut tissue of Diptera, including several disease vectors. Many bacilli and enterobacter such as *Lactobacillus* and *Pantoea* have been identified from the midgut and other tissues of the fruit fly *Drosophila melanogaster* [1]. In field collected *Anopheles stephensi*, *Anopheles gambiae*, and *Aedes aegypti* from laboratory colonies, a number of G+ and G- bacteria were identified [2–5]. Among G-, *Pantoea agglomerans* was also the most common genus identified from all cultivable bacteria in both male and female *Aedes albopictus* collected from two out of four sites in Madagascar [6]. In sand flies, several studies have focused on regional and potential species-specific variability in the microbial community of both *Phlebotomus* and *Lutzomyia* species. *P*. *agglomerans* and *Bacillus* spp. were commonly found in both natural and laboratory-reared sand fly populations. *Bacillus subtilis* and non-*Pantoea* members of the Enterobacter family were shown to be present in populations of *Phlebotomus papatasi* from, India, Turkey, Tunisia, and Egypt [7,8]. *Bacillus* spp, *Serratia marcescens*, and *P*. *agglomerans* were identified in natural and laboratory populations of adult *Lutzomyia longipalpis* [9–12]. In laboratory colonies, Bacilli and Enterobacter were also identified in the larval stages of *L*. *longipalpis* [13,14]. However, to date, most studies were focused on microorganisms present in the adult sand fly with little or no attention paid to physiological effects brought about by these microorganisms on developing stages. Here, we investigated the effects of EGFP-expressing *B*. *subtilis* (*Bs*) or GFP-expressing *P*. *agglomerans* (*Pa*) on the midgut innate immunity and epithelial homeostasis of 3^rd^ instar larvae of *L*. *longipalpis*. Additionally, we determined that the distribution of these bacteria within the midgut of sand fly larvae is in part driven by the gut pH, and demonstrate a cytotoxic effect on the midgut epithelium by infection with *Pa*. Strikingly, we show evidence suggesting a differential and suppressive response to infection with respect to G+ and G- bacteria, likely influenced by the gene encoding the negative regulator of immunity, *"poor immune response upon knock-in*” or *Pirk*. Up-regulation of *Pirk* transcript level leads to a significant depletion of the transcripts encoding the antimicrobial peptide *Attacin* and the immunomodulatory peroxidase *IMPer*. With respect to the distribution of these microbes across the length and distal spacing of the midgut, G+ *Bs* were observed throughout the entire alimentary canal in larvae, whereas G- *Pa* were found primarily in the posterior midgut. Our results strongly suggest that the range of pHs present within the sand fly larval gut likely is a driving force defining the ability of certain bacteria such as *P*. *agglomerans* to infect areas of the gut. The results presented here may have implications beyond the sand fly system and may explain how the distribution (and possibly colonization) of bacteria and other microbes may occur within the guts of insects.

## Materials and Methods

### Sand fly colony maintenance

*L*. *longipalpis* (Jacobina strain–LLJB) were the colony maintained in the Department of Entomology, Kansas State University. Larvae were maintained in 250 or 500 ml plastic jars (Nalgene) with an approximately 2 cm-thick bed made of dental plaster (Schein), and fed on either larval chow (a mixture of 50% rabbit droppings and 50% rabbit food) or on 1.5% agar in LB, with or without the (E)GFP-expressing bacteria (see below).

### Culturing and feeding of EGFP-expressing *B*. *subtilis* and GFP-expressing *P*. *agglomerans*

EGFP-expressing *B*. *subtilis* (strain 1012 transformed with plasmid pAD43-25, obtained from the Bacillus Genetic Stock Center) and GFP-expressing *P*. *agglomerans* (strain EPA-E325 transformed with plasmid pT-3078-5, a gift from Dr. David Lampe) were cultured at 30°C overnight in LB medium supplemented with 5 μg/ml chloramphenicol (Alfa Aesar, A Jonson Mattey Co.) or with 50 μg/ml Carbenicillin (Teknova), respectively. Bacterial cultures were centrifuged at 2500 rpm for 20 minutes at room temperature and the pellets were washed twice with 1X PBS. Bacteria were then suspended in PBS for a final concentration of 10^9^ bacteria in 50-to-80 µl that was spread on a plate containing a thin layer (2–3 mm thick) of LB-agar (no antibiotics) and grown overnight. The following day, fluorescence of the bacterial lawn was confirmed and the LB-agar was cut into 3-to-5 mm^2^ pieces to be fed to early L3 *L*. *longipalpis* larvae (depicted in S1 Fig), and lawns were replaced every 48 h. Third instar larvae were used to maximize food intake as L3s feed more and for a longer period of time than L4. Prior to feeding on the LB-agar with *Bs* or *Pa*, all larvae were starved for 6 to 10 hours to allow for excretion of midgut contents, and rinsed in sterile water. As controls, L3 larvae were fed on LB-agar plus 5 mM Paraquat, an insecticide that strongly induces apoptosis [15], and on LB-agar plus Kanamycin (50 µg/ml) (we were unable to use plain LB-agar due to contamination). Larvae fed *ad libitum* for up to 48 h at 26°C and 80% humidity, with a 12:12 h light-dark cycle. Groups of (n = 20) larvae were collected at 12, 24, 36, and 48 h post feeding with three biological replicates. Food intake was determined by examining each larva under a dissecting microscope (10X). All larval feedings were done according to feeding groups using 500 ml Nalgene pots with a 2–3 cm layer of dental cement.

Alternatively, larvae were fed for 12 h on bacteria-containing LB-agar and transferred to pots with plain LB-agar (no bacteria and no antibiotics). Larvae in groups of 3 to 5 were collected every 3 h and assessed for GFP signal using a Zeiss confocal LSM microscope. CFUs were also measured for larvae collected at 12 h and 24 h, by surface sterilizing each larva, dissecting and grinding each whole gut using a hand-held homogenizer in 60 µl 1X PBS, and plating the homogenate on selective media (5 µg/ml chloramphenicol for *B*. *subtilis* or 50 µg/ml carbenicillin for *P*. *agglomerans*) and incubating at 28ºC to 30ºC.

To assess for any effects of diet on midgut development, the length and width of the larval midgut were measured using the LSM 510 using the software ZEISS LSM Image Browser (Zeiss International). Midgut length was determined by measuring from the beginning of the anterior midgut to the posterior region of the midgut. Width measurements were obtained from three regions of each midgut: the anterior (ant), the middle (mid), and the posterior (pos) regions.

### Immunocytochemistry

Whole guts from *L*. *longipalpis* L3 larvae were dissected (n = 3) from three separate treatments of L3 into PBS and fixed for 20 minutes at room temperature with 4% paraformaldehyde in PBS. Tissues were washed 4 times for 30 minutes with PBS containing 0.3% Triton X-100 (PBST), then blocked with PBS containing 1% bovine serum albumin for 30 minutes at room temperature. Tissues were then incubated overnight at 4°C with primary antibodies for rabbit anti cleaved caspase3 (Cell Signaling Technology) diluted 1:500 in PBST. Tissues were washed 3 times for 30 minutes with PBST, and incubated overnight at 4°C with Alexa Fluor® 594 goat anti-rabbit (Invitrogen) diluted 1:1000 in PBST. Tissues were washed 3 times for 30 minutes with PBST, and nuclei were stained for 5 minutes with 10 µg/mL of DAPI (Invitrogen). Samples were mounted in Vectashield® (Vector Laboratories) anti-photo bleaching reagent, and images were obtained with a LSM 510 confocal microscope using the software ZEISS LSM Image Browser. In addition, measurements of larval guts length and width were obtained for each feeding treatment using the LSM510 confocal microscope. For microscopy, entire alimentary canals were used for viewing clarity, whereas for gene expression analyses, all hindguts were removed prior to RNA isolation of larvae.

### Larval midgut pH

We assessed the pH within the midgut by feeding *L*. *longipalpis* L3 larvae with LB-agar containing 0.4% of the pH indicators Bromothymol blue and Phenol red. LB-agar medium adjusted to pH 7 added prior to sterilization. Each indicator agar medium was fed to a group to 50 L3 larvae following six hours of food deprivation. Feeding of the larvae was performed by placing the larvae and fragments of approximately 3–5 mm^2^ of the dye-containing agar inside a 500 ml Nalgene pot with a 1 cm layer of plaster, maintained at room temperature and with a relative humidity of 80–85%. Larvae were allowed to feed *ad libitum* for 20 hours. The pH indicator dyes were visualized through the translucent cuticle of the larvae. The pH inside the larval gut was determined by comparing the color and intensity shown within the gut with those from 0.4% solutions of both dyes made in 8 ml LB medium (with one added drop of chloroform to prevent bacterial growth), and with pH ranging from pH 4 to 10 in 0.5 increments. Larvae were also fed on 0.1 and 0.4% thymol blue.

### Effect of pH on *in vitro* growth of EGFP-expressing *B subtilis* and GFP-expressing *P*. *agglomerans*

Overnight cultures of fluorescent *Bs* and *Pa* were diluted to OD_600_ = 0.1 and further diluted 1:10^4^ prior to plating onto LB-agar plates supplemented with either 50 µg/ml of carbenicillin (CAR) or 5 µg/ml of chloramphenicol (CAM) for selection of *Pa* or *Bs*, respectively. The pH of plates ranged from pH 6 to 9.5 in 0.5 increments. Plates were incubated overnight at 37°C for *Bs*, and at room temperature for *Pa*, and each experiment was performed in triplicate, and repeated twice. The following day bacterial colonies were counted, and colonies growing at each pH were observed under fluorescent microscope. One-way ANOVA with a Tukey test was performed to determine differences between pH.

### RNA extraction and reverse transcription

Midguts from *L*. *longipalpis* L3 larvae were dissected under a stereoscope microscope in Hyclone (Thermo Scientific) phosphate buffered saline (PBS) at 12, 24, and 36 h post feeding in either the *Bs* or *Pa*. Sterile, 1.5% agar in LB was used as feeding control. Total RNA was isolated from pools of 20 midguts using TRIzol (Invitrogen). For each group of larval midguts, RNA isolation was done in triplicate. RNA quality was assessed by electrophoresis on 1% agarose-5% formaldehyde in 1x MOPS, and stored at -80°C. First strand cDNA synthesis was conducted using Superscript III reverse transcription kit (Invitrogen) as described [16].

### Quantitative Real Time PCR

mRNA levels were quantified with iQ SYBER Green Supermix (Bio-Rad) using 95°C melting, 57°C annealing, and 72°C extension temperature for 40 cycles using a Realplex^4^ Master cycler (Eppendorf). Relative fold changes were assessed using the ∆∆Ct method [16,17], and calibrated against the expression observed for same stage larvae fed on the plain LB-agar control. Sequences for *Attacin* (*Att*), *IMPer*, *Vein*, *Domeless*, *IMD*, *Pirk*, *USP36*, and *Duox* were obtained using the tBLASTN algorithm from corresponding annotated sequences found in *D*. *melanogaster* blasted against *L*. *longipalpis* contigs. Predicted full-length transcripts were made using GENSCAN (http://genes.mit.edu/GENSCAN.html), and primers sequences were generated using Primer3 (http://biotools.umassmed.edu/bioapps/primer3_www.cgi). Primers for RPS6 were previously described in [16]. Primers for *Def1* were based on the sequences described in an earlier study [11]. All other primers used in this study were designed from gene sequences in the NCBI database and their accession numbers are as follows: IMPer, AJWK01035414.1; DUOX, AJWK01035414.1; IMD, AJWK01008032.1; Domeless, AJWK01008028.1; Attacin, AJWK01017071.1; Pirk, AJWK01015539.1; USP36, AJWK01027563.1; and Vein, AJWK01005322.1.All primer sequences used in this study are summarized in S1 Table.

## Results

### *Bs* and *Pa* localize to different regions of the sand fly larvae midgut

**Fig 1:**
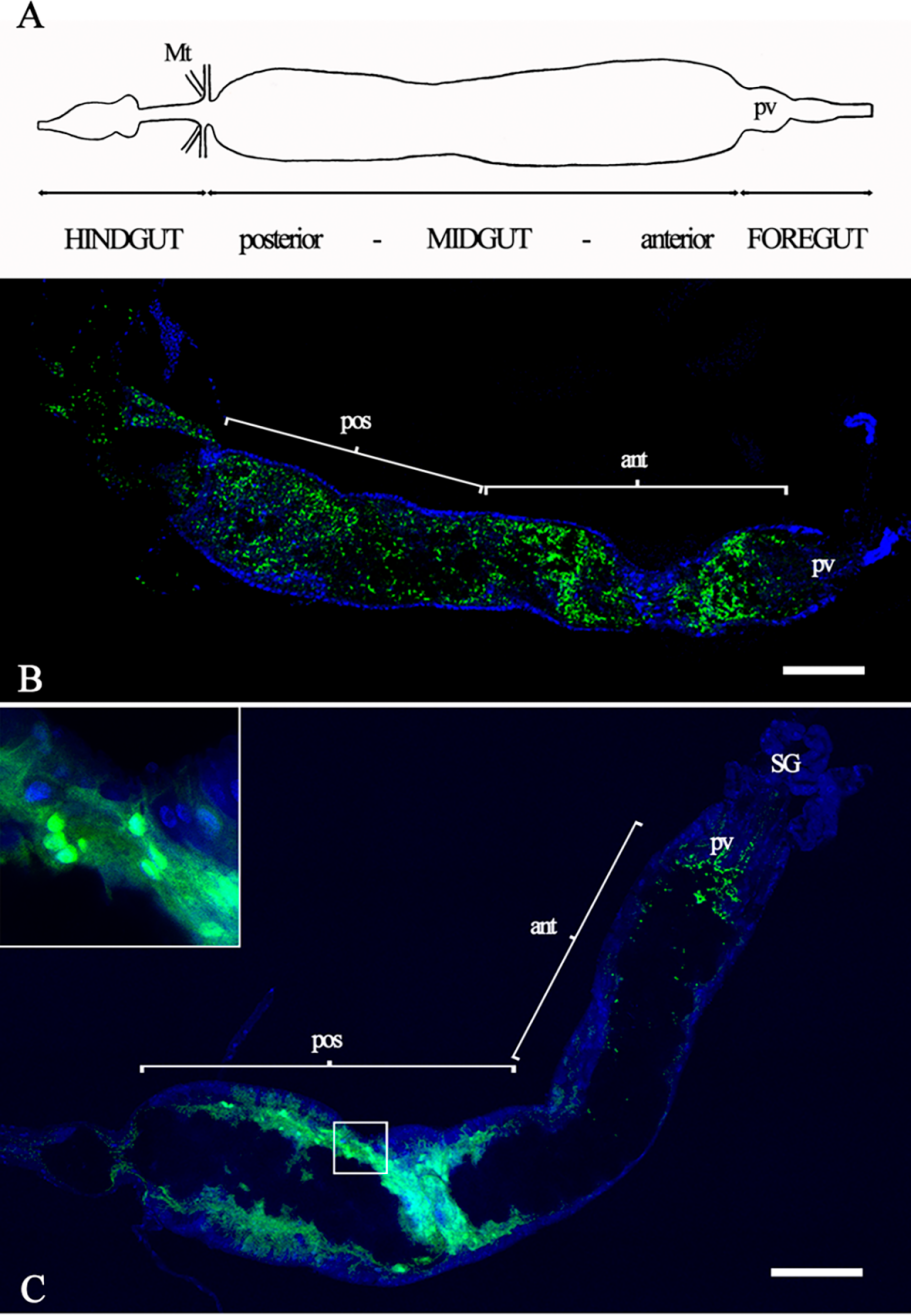
Infection of sand fly larva midgut by *B*. *subtilis* and *P*. *agglomerans*. EGFP- or GFP-expressing *Bs* and *Pa* were grown on LB-agar plates with selective media and fed to 3^rd^ instar sand fly larvae. Larvae guts were dissected and assessed for the distribution of each bacterium. In A, a schematic representation of the sand fly larval gut. Ingested food is moved from right (proventriculus–pv) to left, towards to posterior midgut and hindgut. Confocal images (1024x1024 per tile pixel resolution) of the distribution of EGFP-expressing *Bs*-infected and GFP-expressing *Pa*-infected midguts are shown in panels B and C. Posterior (pos) and anterior (ant) portions of midguts are indicated. SG, salivary glands. Inset in 1C: blow up of area of gut delineated by a rectangle (asterisk) showing biofilm formed in *Pa* infection. Bars = 100 µm.

A scheme representing the anatomy of *L*. *longipalpis* sand fly 3^rd^ instar (L3) gut is depicted in Fig 1A. Following continuous feeding of LB-agar containing EGFP-expressing *Bs* to larvae, a pervasive signal was found across the entire length of the midgut for infected insects as depicted by the fluorescent signal of full length images of the gut (Fig 1B and S2A Fig and S1 Video). However, when the GFP-expressing *Pa* was fed to larvae in a similar manner the bacteria were mostly localized to a narrow area of the posterior portion of the midgut (Fig 1C and S2B Fig), and were only found on the apical surface of the midgut lumen (S2 Video). Also observed were areas of higher intensity GFP signal in *Pa*-infected guts, suggesting the presence of biofilm (Fig 1C). A less pronounced GFP signal in *Pa* fed was also observed in the proventriculus of the gut (Fig 1C). Infection rates as determined by a qualitative assessment of the GFP signal within the midgut of the larvae following continuous feeding are described in Table 1.

**Table 1:** *P*. *agglomerans* and *B*. *subtilis* infection rates.

GFP positive signal		
Time post infection	*P*. *agglomerans*	*B*. *subtilis*
12 h	9 of 14 (64%)	14 of 15 (93%)
24 h	0 of 9	15 of 15 (100%)
36 h	1 of 9 (11%)	7 of 7 (100%)
48 h	0 of 9	3 of 8 (37.5%)

*P*. *agglomerans* and *B*. *subtilis* were continuously fed to sand fly larvae. At time points indicated (left column), the infection rates in sand fly larvae were determined by a qualitative assessment (presence or absence) of GFP signal using a Zeiss 510 confocal microscope.

**Fig 2:**
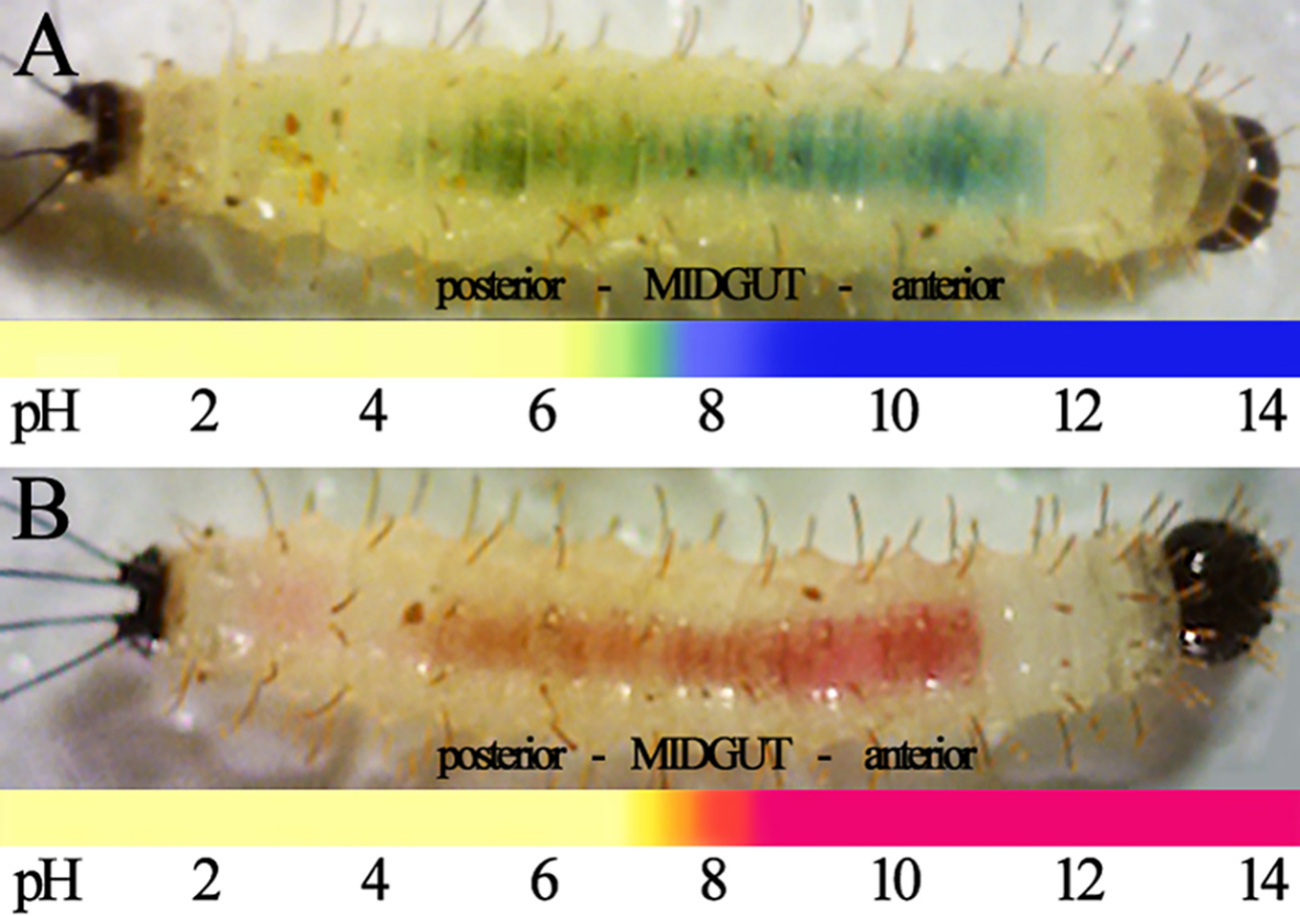
pH of the sand fly larval gut. Third instar sand fly larvae were fed with pH indicators Bromothymol blue (A) and Phenol red (B). Shown is the distribution of each indicator within live sand fly larvae guts, with the predicted pH for each area of the gut indicated. Live 3^rd^ instar sand fly larvae are shown from left (posterior or caudal setae) to right (anterior or head).

To further investigate what could be driving such a distinct microbial distribution, we assessed the gut pH range within larvae *in vivo*, and compared it to pH growth assays for the two GFP-expressing bacteria *in vitro*. LB-agar supplemented with pH indicators Bromothymol blue or Phenol red were fed to larvae, and the color gradient generated was visible through the translucent cuticle of larvae using light microscopy. Intensity of colors varied between larvae due to the initial ingestion time, load size, and bolus movement across the gut. Fifteen larvae of each treatment were compared to the pH references. The Bromothymol blue dye has a range of pH from 6 to 7.6, and Phenol red has a range of 6.8 to 8.4. Previous results on *L*. *longipalpis* larval gut pH indicated a basic pH >9 in the anterior portion and an more acidic pH >6.5 in the posterior portion of the midgut [18]. Larvae fed on thymol blue (at concentrations of 0.4% and 0.1%) displayed a green-colored gradient that was not easily distinguishable between pHs 8.5 to 9.5 through the larval cuticle. Two other pH indicators, alizarine yellow and thymolphthalein, were considered during our studies. However, because of our choice of using whole larva, the output colors of these pH indicators were not suitable for visualization through the insect cuticle. The results shown in Fig 2 confirm such a pH gradient in the *L*. *longipalpis* larvae, clearly pointing to a basic pH for the anterior part of the midgut, including the proventriculus (PV), and an acidic pH in the posterior part of the midgut.

**Fig 3:**
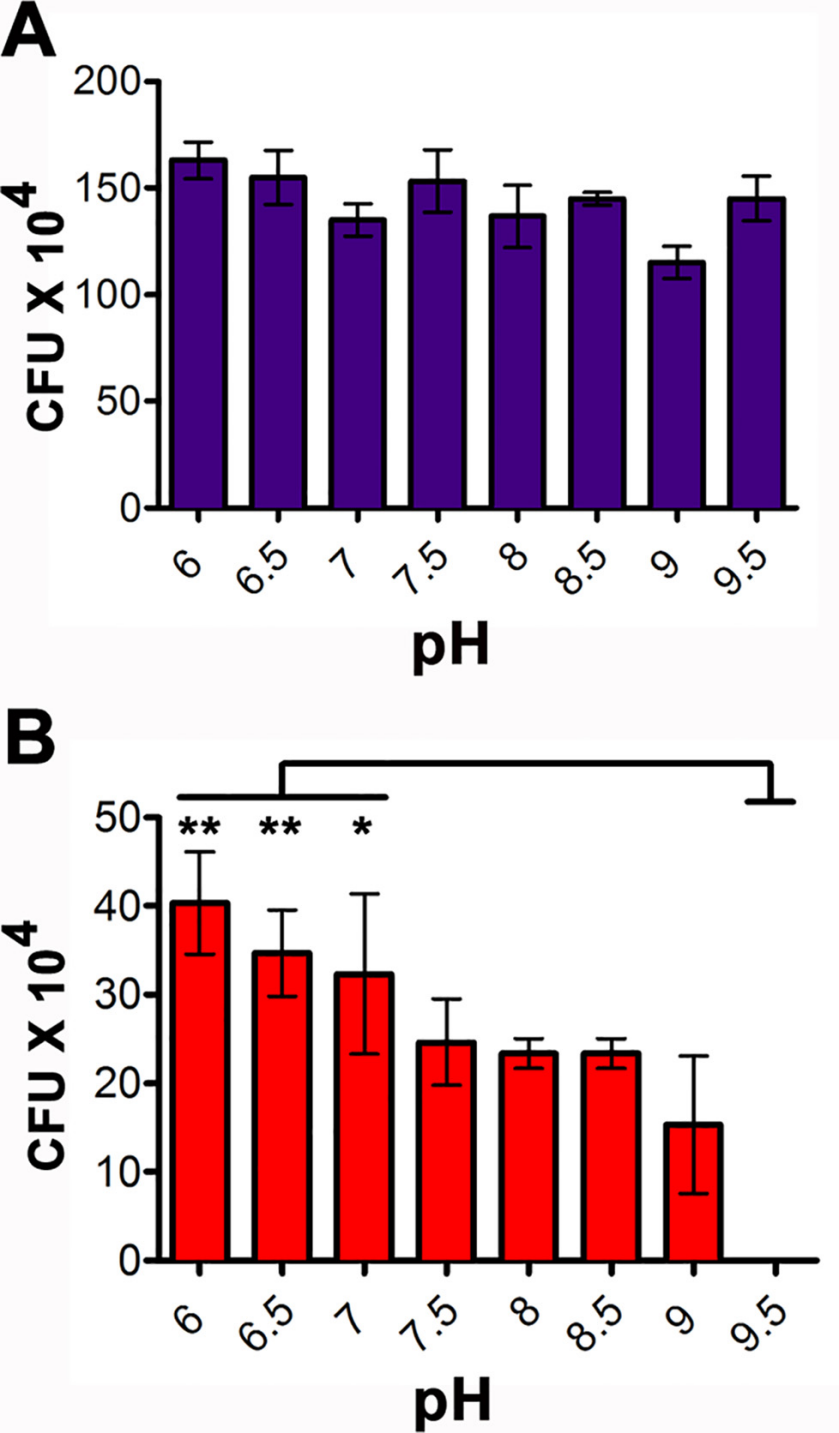
Effect of pH on in vitro growth of *B*. *subtilis* and *P*. *agglomerans*. Cultured bacteria were grown on LB-agar plates of pH varying from 6–9.5. The colony forming units are measured for *Bs* (A) and *Pa* (B). One way ANOVA, with a post hoc Tukey test, was performed to assess significance. pHs 6, 6.5, and 7 were statistically different than pH 9.5 (P<0.01 for pH 6 and 6.6; and P<0.05 for pH 7).

Both *Bs* and *Pa* bacteria were grown on antibiotic supplemented LB-agar plates with pH ranging from 6 to 9.5. CFU counts were obtained in triplicate to assess the viability of the two strains at different pH (Fig 3A). Colonies of EGFP-expressing *Bs* did not show a significant difference in CFU counts at any given pH. Additionally, the colony size for *Bs* fed was smaller at low (6–6.5), and high (9.5) pH. However, *Pa* showed a significant difference in CFU counts at pH ranging from 6–7 with respect to pH 9.5 (Fig 3B). Also, at pH 8–9 colony size and fluorescent intensity began to decrease, and by pH 9.5 there was no visible growth.

Following the continuous feeding experiment described above, we tested whether similar results could be obtained by feeding larvae once with either *Bs* or *Pa*. Larvae were fed for 12 h on LB-agar with the respective bacteria and transferred to pots with fresh LB-agar (no antibiotics). *Pa* bacteria were cleared by 21 h after infection, whereas *Bs* were cleared by 24 h (Table 2). CFU counts (Table 3) were generally in agreement with the results observed for the GFP signals assessed. No bacteria from pupae were able to be re-cultured. Interestingly, sand fly larvae may become cannibalistic when food is scarce. We also noticed that the *Pa*-infected larvae appeared to rely on cannibalism more frequently than *Bs*-infected.

**Table 2:** Persistence of bacterial infection between continuous and non-continuous feedings.

	GFP positive signal			
	Continuous feeding		Non-continuous feeding	
Time	*P*. *agglomerans*	*B*. *subtilis*	*P*. *agglomerans*	*B*. *subtilis*
12 h	8 of 10*	10 of 10*	-*	*-
15 h	-	-	1 of 5	4 of 5
18 h	-	-	1 of 5	1 of 5
21 h	-	-	0 of 5	3 of 5
24 h	1 of 5	5 of 5	0 of 4	0 of 5
27 h	-	-	0 of 5	0 of 5
30 h	-	-	0 of 5	0 of 5
36 h	1 of 5	4 of 4	0 of 3	0 of 2
42 h	-	-	0 of 5	0 of 5
48 h	0 of 5	3 of 5	0 of 5	0 of 5

Persistence of bacterial infection in sand fly larvae was determined by a qualitative assessment (presence or absence) of GFP signal using a Zeiss 510 confocal microscope. GFP positive signal observed for larvae fed for up to 48 h on bacterial lawns (continuous feeding), of for larvae fed 12 h on bacterial lawn of each bacteria, and transferred to plain LB-agar (non-continuous feeding). Larvae were collected at different time points and the midguts were dissected and prepared for confocal analyses as indicated. Results observed for the continuous and non-continuous feeding are shown as number of larvae displaying a GFP signal per total larval guts.

*As larvae in both groups fed for 12h, there is no difference between continuous and non-continuous for that time point; all larvae were grouped into the continuous feeding group.

**Table 3:** Colony forming units (CFU) from continuous and non-continuous fed larvae.

	Continuous feeding		Non-continuous feeding	
Time	*P*. *agglomerans*	*B*. *subtilis*	*P*. *agglomerans*	*B*. *subtilis*
12 h	0–48 (n = 5) (10.8 ± 20.9)	30–2600 (n = 5) (647.6 ± 1095.6)	-	-
24 h	4–6 (n = 3) (6.0 ± 2.0)	0–480 (n = 3) (330.2 ± 286.2)	0–190^†^ (n = 3) (63.3 ± 109.7)	0 (n = 3)

Range (AVG±Stdev) distribution of CFUs present in gut larva at 12 h (n = 5) and 24 h (n = 3) after continuous or non-continuous feeding. In continuous feeding, larvae were fed on LB-agar plus bacteria for 24 h; for non-continuous feeding, larvae were fed on LB-agar plus bacteria for 12 h and transferred to LB-agar. Guts were dissected from surface sterilized larvae and homogenized in 60 µl of 1X PBS followed by plating on selective LB media. After overnight incubation at 37°C plates were scored for the presence of colonies assessed according to morphology and GFP signal. Results shown are representative of three experiments.

†CFUs present in *Pa*-fed larva at 24 h were likely due to cannibalism observed in this group.

### Infection of the sand fly larvae midgut by *Pa* or *Bs* leads to differential damage of the larval midgut epithelium

**Fig 4:**
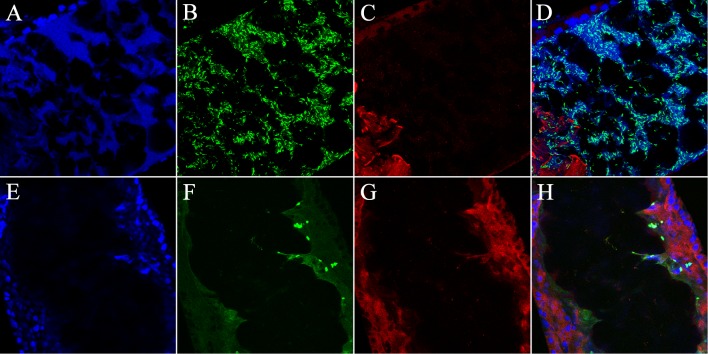
Confocal images of *B*. *subtilis* and *P*. *agglomerans* infection of sand fly larvae midguts. Anterior midgut image12h post feeding, EGFP-expressing *Bs* is able to infect the entire length of the midgut. A) DAPI staining; B) Shows *Bs* distributed throughout the anterior larval gut; C) Immuno-staining for cleaved caspase3 along the lumen of the midgut (the bright red staining in the bottom corner was due to auto-fluorescence associated with remnants of the colony chow previously fed to the larva). D) Merge. 12h post infection with GFP-expressing *Pa* localized to the posterior region of the midgut epithelium and induces apoptotic activity. E) DAPI staining depicting the midgut epithelium. F) Shows *Pa* localized on the apical portion of the lumen of the midgut. G) Immuno-staining for cleaved caspase3 along the lumen of the midgut depicting high levels of caspase3 activity. H) Merge. Bars = 50 µm.

*Bs* and *Pa* were assessed for their ability to infect the sand fly L3 larvae midgut following feeding, and their effect on induction of apoptosis. A monoclonal antibody targeting the cleaved caspase3 was used as an immunocytochemical marker to identify epithelial cells undergoing caspase-dependent programmed cell death, and to assess the integrity of the midgut. This antibody was used previously in Drosophila to detect c-Jun N-terminal kinase (JNK) pathway activation in response to infection [19]. In order to test if this was a viable approach, we fed larvae with LB-agar supplemented with the apoptosis inducer Paraquat, and compared its effects to larvae fed on LB-agar alone. The LB-agar fed larvae displayed a well-defined midgut epithelium with little background staining for active caspase3 (S3A Fig). In contrast, larvae fed on LB-agar supplemented with Paraquat showed midgut epithelia with significant loss of integrity, that were also severely flattened after mounting on the slide with reduced luminal space detectable by looking at nuclei alone (S3B Fig). The apoptotic effect of the Paraquat was further confirmed by the presence of a large population of cells showing heavy cytoplasmic specific staining for caspase3 (S3C and S3D Fig). After 12h of infection with *Bs* an extensive amount of luminal nucleic material is observed using DAPI staining (Fig 4A), and a massive infection can be seen in Fig 4B. Very little background caspase staining is observed in *Bs*-fed compared to the Paraquat treated controls (Fig 4C and 4D, and S3 Fig). In contrast, when *Pa* was used for infection, the Gram-negative bacteria induced staining comparable to that of the Paraquat control (Fig 4G and 4H and S3 Fig). However, we did not observe a similar breakdown in midgut superstructure.

### Effect of feeding agar on larval development

Effects of feeding agar to the developing sand fly L3 larvae were assessed by comparing the length of the whole midgut and the width of three areas within the midgut to those obtained from larvae raised on regular larvae food (50% rabbit feces + 50% rabbit food). The same parameters were also measured from guts of *Pa* and *Bs* infected larvae. A significant difference was found for midgut length when comparing regular sand fly larval food and the agar fed, except at 48 h. For the three measurements of gut width (anterior, middle and posterior), significant differences were only observed between regular food and agar (S4 Fig).

### Bacterial feeding leads to a differential expression profiles in sand fly larvae

We compared the mRNA expression profiles of nine genes involved in various physiological processes ranging from innate immunity, homeostasis, and epithelial regeneration in midguts of *L*. *longipalpis* L3 larvae fed on *Bs* and *Pa* bacteria. Among the transcripts assessed were *Att*, *Def1*, *Duox*, *IMPer*, *Vein*, *Domeless*, *IMD*, *Pirk*, and *USP36*. Results from qRT-PCR indicate that, compared to control fed, larvae fed on agar containing either *Bs* or *Pa* showed significant difference in expression for a number of genes analyzed.

**Fig 5:**
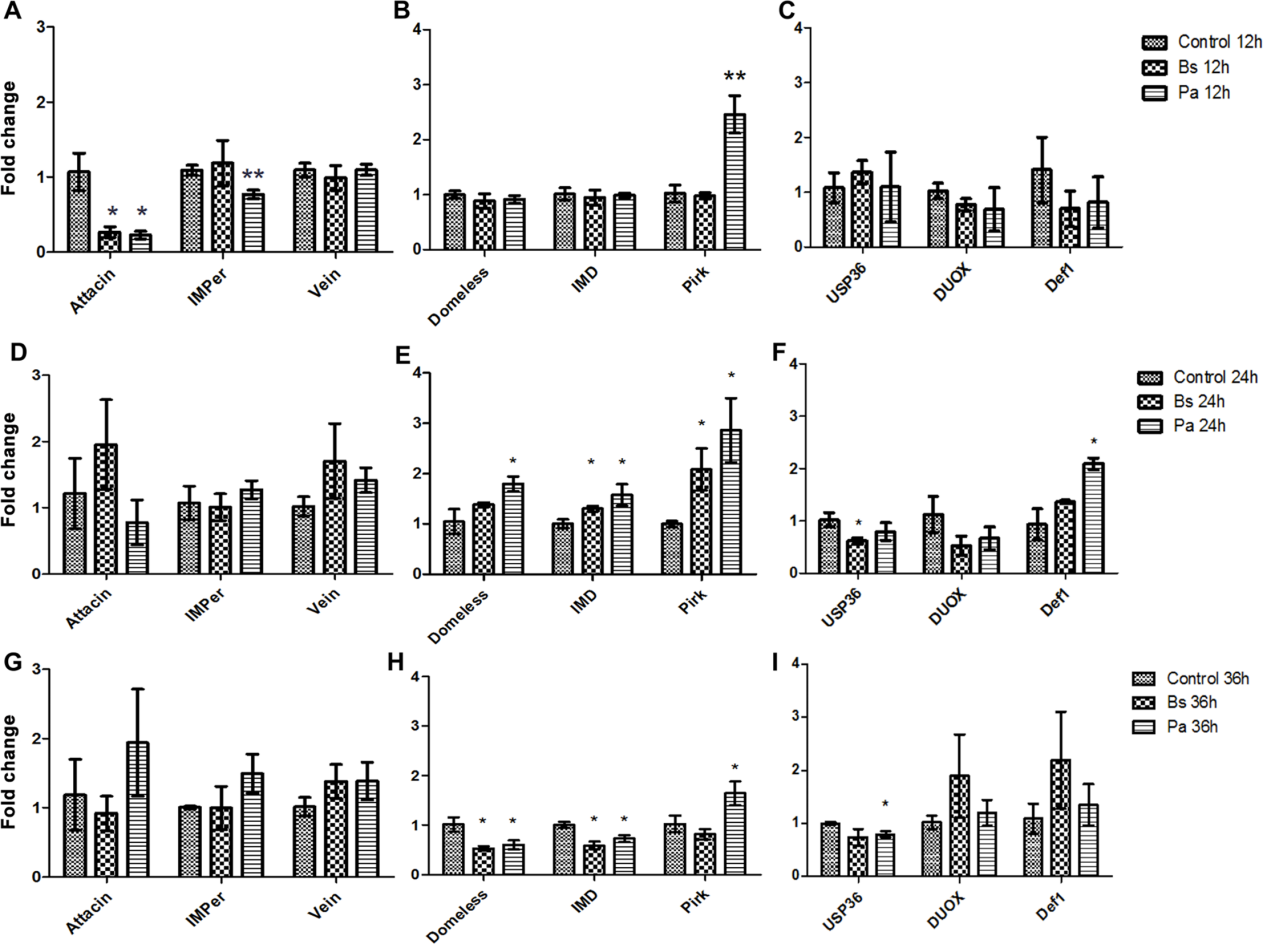
mRNA expression profiles of 3^rd^ instar *L*. *longipalpis* larvae post infection with *B*. *subtilis* and *P*. *agglomerans*. *B*. *subtilis* (*Bs*) or *P*. *agglomerans* (*Pa*) bacteria were fed to larvae and the expression of selected transcripts relative to agar fed control larvae was assessed. Expression profiles were obtained for the effector molecules *Att* and *IMPer*, in addition to the epithelial growth factor *Vein* are shown in A. D, and G, Expression profiles for the JAK/STAT receptor *Domeless*, the transcription factor for immunodeficiency *IMD*, and the negative regulator of IMD pathway *Pirk* are shown in are shown in B, E and H. Expression profiles for the E3 Ubiquitin ligase associated with *IMD*, and the effector molecules dual oxidase *DUOX* and *Def1*are shown in C, F, and I. Following bacterial feeding, total RNA was obtained at 12 h (A, B, and C), at 24 h (D, E, and F), and at 36 h (G, H, and I) post infection (PI). All Ct values were normalized to the ribosomal protein S6 (RPS6). Error bars are represented as the standard deviation determined from three biological replicates each with n = 20 midguts. Significance was determined using student t-tests. * denotes P<0.05 and ** denotes P<0.01. Y-axis, fold change.

At 12 h post infection, transcript levels for *Att* were downregulated by nearly 75% for both *Bs* and *Pa* infected larvae, and *IMPer* was downregulated by 25% in *Pa* fed (Fig 5A). *Pirk* showed a 2.5-fold change (~150% increase) in expression following infection with *Pa* (Fig 5B).

At 24 h post infection, *Domeless* and *IMD* followed a similar profile and were upregulated in both infections, albeit *Domeless* was not significantly different between the control and *Bs* fed. *Pirk* was also upregulated at in both bacterial infections, and with a profile that also was similar to *Domeless* and *IMD* (Fig 5E). *USP36* was downregulated only in *Bs* infection, and *Def1* was upregulated only in *Pa* (Fig 5F).

At 36 h post infection, *Domeless* and *IMD* again displayed similar profiles, however both were downregulated in comparison to control (Fig 5H). For *Pirk*, whereas expression if *Bs* fed returned to control levels, in *Pa* infection it remained significantly higher (nearly 2-fold) (Fig 5H). Finally, *USP36* expression was reduced by roughly 10% in both infections, but for *Bs* the significance was 0.078 (Fig 5I).

## Discussion

In insects, gut bacteria have been shown to significantly contribute to nutrition, modulation of the immune response, and protection from parasites and other pathogens. The insect gut varies greatly in terms of morphology and physicochemical properties, and these may influence the distribution and structure of the microbial community in the gut. During development, sand fly larvae are exposed to a wide variety of soil bacteria and other microorganisms that are able to colonize the insect gut [7–14,20]). However, as we are aware, no studies have focused on the mechanisms by which bacteria are able to develop within the sand fly gut, or the types of specific responses induced by the colonization. Here, we assessed the ability of two bacteria previously identified from the guts of insects, including sand flies, to infect the gut of sand fly larvae, and investigated the specific responses (innate immunity, epithelia regeneration, homeostasis) induced by these bacteria.

When fed to *L*. *longipalpis* larvae, EGFP-expressing *Bs* bacteria were distributed throughout the entirety of the alimentary canal, mainly within the peritrophic matrix and along the lumen. In contrast, GFP-expressing *Pa* bacteria were mostly localized to the posterior midgut, and only at the apical surface (although we did observe GFP signal for *Pa* at the proventriculus of the gut, it is possible that these bacteria had not yet been killed by the alkaline conditions). We speculated that this phenomenon must be driven by pH and/or by specific cell types that line the midgut lumen. A pH driven effect on the distribution of bacteria in the sand fly larval gut was suggested by the use of pH indicators. It has been previously determined in sand fly larvae that the pH of the midgut is highly alkaline at the anterior portion and decreases towards the posterior region [18]. Our approach allowed us to confirm the location within the larval gut wherein the pH ranges between 6 and 7, which also coincides with the infection of the Gram-negative *Pa*. These results were indirectly confirmed by *in vitro* growth assays obtained for *Pa* in which these bacteria clearly favor a pH in the range of 6-to-7. The ability of *Bs* to sporulate under unfavorable growth conditions may have further contributed to its distribution along the gut of the larvae.

In addition, we observed a marked difference between the rates and the persistence of infections of *Pa* and *Bs* in sand fly larvae. Following continuous feeding on bacterial lawn, most of the *Pa* bacteria are cleared within 24 h whereas infection with *Bs* remained for up to 48 h. However, if the bacterial lawn is replaced at 48 h during continuous feeding, larvae do re-infect. In contrast, with the non-continuous feeding on the bacterial lawn led to clearing of *Pa* by 21 h and *Bs* by 24 h. Hence, the data indicate that the sand fly larvae are able to clear bacterial infection, by the activation of the antimicrobial immune response, if exposure is not maintained. Another possibility is that the loss of *Pa* and *Bs* during non-continuous feeding may also be caused by competition with other microorganisms present in the larval gut. And in spite of differences known to exist in the half-lives of GFP and EGFP proteins [21,22], the CFU counts reported support the clearing of *Pa* from the midgut during loss of GFP signal.

Using a caspase3 antibody [19,23] to detect apoptotic activity in *L*. *longipalpis*, we also were able to clearly identify differences between *Bs* and *Pa* infection of the sand fly larval gut. The microscopy data strongly suggest that only *Pa* induces caspase activity within the midgut of the sand fly, while *Bs* causes little to no staining.

Quantitative RT-PCR analyses were used to assess changes in the expression profiles of nine selected genes chosen based on their roles in insect midgut immunity and homeostasis. Related to midgut immunity, selected genes included those coding for effector molecules such as the antimicrobial peptides (AMPs) attacin (*Att*) and defensin (*Def1*), as well as *Duox* and *IMPer*. Also included in this category was the immunodeficiency regulatory gene encoding *" poor immune response upon knock-in”* or *Pirk*.

Attacin has long been implicated in bacteria killing from a number of studies pertaining to its role in innate immunity [24]. *Def1* was shown to be upregulated in adult *L*. *longipalpis* after bacterial challenge [11]. It has been shown that Defensin A, acting in concert with Cecropin A, blocks *Plasmodium* transmission in *A*. *aegypti* [25]. The effector molecules Duox and IMPer have been demonstrated to have effects on the midgut peritrophic matrix structure and parasite killing in *A*. *gambiae* mosquitoes [26–28]. For Def1, Att, DUOX, and IMPer, there are multiple studies suggesting that these effector molecules are regulated by the immunodeficiency pathway [27,29,30]. Pirk has been previously shown to be a negative regulator of IMD activity [31,32]. While Pirk acts to suppress IMD at the level of signal transduction, Caspar negatively regulates IMD at the level of transcription. Studies in *A*. *gambiae* implicate the knockdown of *IMD* in increased infectivity of mosquitoes [33,34]. In sand flies, *Caspar* knockdown led to a decrease in *Leishmania mexicana* load in *L*. *longipalpis* [12].

*Domeless*, *Vein*, and *USP36* were selected based on their roles in pathways related to innate immunity to midgut regeneration. When the innate immune response is activated in the midgut, there are associated energy costs and damage to healthy epithelial cells that can negatively affect the insect. Artificially activating ROS production in *A*. *stephensi* led to reduction in infective lifespan, and deleterious effects associated with mitochondria [35]. Domeless is a receptor in the JAK/STAT pathway that is crucial for recognizing damage to healthy epithelial cells. JAK/STAT signaling reaches intestinal stem cells (ISCs) and enteroblasts (EBs) leading to the secretion of an epidermal growth factor (Vein) ending in regeneration of midgut epithelia via proliferation and differentiation of ISCs and EBs [19,36–38]. Additionally, the deubiquitinating enzyme USP36 is a negative regulator of IMD and provides a route of cross-talk between IMD and JAK/STAT pathways [39,40]. USP36 is also involved in controlling selective autophagy [40].

Our results suggest that *Pirk* may be acting to suppress *Att* and *IMPer* activity at 12 h post infection for *Pa*-infected insects, however, another still unidentified mechanism likely is involved in the reduction of *Att* levels for *Bs*-infected. Although *Pirk* was significantly upregulated during *Bs* and *Pa* infection at 24 h, there was also an increase for the immune transcription factor *IMD*. Such *IMD* increase can be linked to an associated upregulation of *Def1* in *Pa* infected, but no significant difference was found in *Def1* for *Bs* infected. The upregulation of *IMD* may be explained by the concomitant down regulation of *USP36* in the *Bs* infected larvae, but not *Pa* infected. Additionally, the nearly two-fold increase of *Domeless* in *Pa* fed larvae suggests the possibility of homeostatic response to damage by the larvae immune response that occurs within the first 12 h of infections as our data have demonstrated.

By 36 h, upregulation of *Pirk* continued in *Pa* infected individuals, but no effect was observed for the expression of the effector molecules. With the downregulation of *USP36*, we expected an upregulation of *IMD*. However, the opposite was detected: *IMD* was downregulated. Interestingly, *Domeless* was also downregulated at 36 h, possibly due to lack (or clearing) of bacteria in the gut as indicated by the non-continuous feeding experiments. It remains to be investigated whether downregulation of *IMD*, when *USP36* levels were also lowered, is associated with increased autophagy during bacterial clearing. The expression analyses data corroborates what was observed with regards to the progress of infection in *Bs* and *Pa*. Of significance, our results indicate that sand fly larvae are able to differentially regulate (or suppress, as the case here) their immune response according to the bacterial challenge they are exposed to.

We have previously shown that feeding different bacteria to *L*. *longipalpis* larvae affects survival and development [14]. In the current study, we demonstrate a selective distribution of bacteria in the larvae driven by gut pH and downstream effects on the larval gut. This provides a link between the type of bacteria infecting (as the case here) or colonizing the gut, physicochemical aspects of the gut, and overall insect health. With regards to mechanisms driving the localization of bacteria, it is also likely that different cell types lining the gut epithelia are involved. In support of this hypothesis, concentrated pockets of *Pa* binding to the posterior end of the larval gut were observed, indicating the presence of a biofilm. However, the presence of a preferred cell type or membrane receptor involved in binding of bacteria cannot be discarded. Gram-negative bacteria are known to form biofilm within the gut of vectors [41]. *P*. *agglomerans* form intestinal biofilms in the Mediterranean fruit fly *Cerititis capitata* [42] that resemble what we observed in sand fly larvae. Taken together, these data suggest a pH-dependent localization or growth of bacteria within the insect midgut previously reported to be a random event [42].

With regards to cell type, Fernandes et al [43] reported the presence of different cell types in *A*. *aegypti* during development and metamorphosis, and a precedent for favored microbial binding was previously demonstrated for *Leishmania major* binding to the midgut epithelial cell lining of the sand fly *P*. *papatasi* [44]. Thus, it is conceivable that at least one of these events may also be involved in dictating the success of bacterial colonization within the sand fly gut. Nevertheless, mechanisms such as autophagy may also play a role in bacterial removal (reviewed in Huang et al. [45]). Further, both *Pa* and *Bs* do not survive metamorphosis.

It is important to note that the agar based feeding system used in our experiments does not replicate the natural conditions faced by sand fly larvae and agar does not provide the necessary nutrition for normal larvae development. As shown by our analyses, the midgut length and width differed significantly between larvae fed on agar versus those fed on regular sand fly larvae chow. However, no differences in such parameters of the midgut morphology were observed between agar fed larvae and the agar plus bacteria fed larvae. Interestingly, differences observed for the midgut parameters tested only lasted until either the agar or the bacterial lawn were replaced. Additionally, larvae were not able to sustain a GFP-positive signal when fed on LB-agar plus bacteria for 12 h and then transferred to plain LB-agar. These data were also supported by CFU counts. Notwithstanding, this method was proven useful for specifically delivering the EGFP- or GFP-expressing bacteria. Similar approaches may be used to deliver selected microbes to sand fly larvae in paratransgenic applications to control sand fly populations [46,47].

In conclusion, this study demonstrates that bacteria selectively infect the sand fly larvae midgut, (possibly) leading to epithelial damage. In addition, the data also point to a modulation of the innate immune response likely controlled by expression of Pirk. We also show for the first time that the insect midgut pH is a factor driving microbial organization of the gut. Our results contribute towards understanding of midgut responses to infections and provide new insights for development of vector control approaches using paratransgenesis.

## Supporting Information

S1 Fig Larval feeding of bacterial lawn. After overnight incubation on LB-agar plus antibiotics, each bacterial lawn was cut and fed to 3^rd^ instar larvae. On the left (A) the larvae are feeding on EGFP-expressing *Bs*, while on the right (B) the larvae are feeding on GFP-expressing *Pa*.
(PDF)

S2 Fig Infection of sand fly larva midgut by *B*. *subtilis* or *P*. *agglomerans*. Larval guts were imaged using a resolution of 512 x 512 (number of pixels per tile). Ingested food is moved from right (proventriculus–pv) to left, towards to posterior midgut and hindgut. EGFP-expressing *Bs*-infected (A) and GFP-expressing *Pa*-infected (B) midguts are shown. Posterior (pos) and anterior (ant) midgut are marked. Arrowheads indicate the separation between midgut and hindgut. Bars = 100 µm.
(PDF)

S3 Fig Paraquat-induced apoptosis in sand fly larvae midguts. Ingestion of Paraquat induces a detectable and systemic apoptotic response in the cytoplasm of midgut epithelial cells 12h post feeding. A) Merge of caspase3 and DAPI stained nuclei for larvae fed only LB-agar medium in the anterior midgut versus B) larvae with LB-agar supplemented with Paraquat visualized with DAPI 12h post infection (anterior midgut). C) Immuno-staining for cleaved caspase3 in Paraquat fed larvae. D) Merge of B and C. Bars = 50 µm.
(PDF)

S4 Fig Changes in sand fly larval gut length and width caused by diet. *L*. *longipalpis* 3^rd^ instar larvae were fed on either regular sand fly larval food (50% rabbit feces + 50% rabbit food), or on LB-agar with or without bacteria, for up to 72 hours. Larval midguts were dissected and measured for length, and width in the anterior, middle, and posterior regions of the gut. Panels: A, midgut length; B, width of the anterior portion of the midgut; C, width of the middle portion of the midgut; D, width of the posterior midgut.
(PDF)

S1 Table Primers list (DOCX)

S1 Video Localization of *B*. *subtilis*. The localization of *Bs* to the entirety of the midgut is depicted in the z-stack video of the gut. The video depicts *Bs* prevalence over the entirety of the midgut contents, beginning from a dorsal view and ending in a ventral view of the midgut epithelium.
(AVI)

S2 Video Apical localization of *P*. *agglomerans* on midgut epithelia. The localization of *Pa* to the apical surface of the midgut lumen is shown in the z-stack video of the gut. The video depicts *Pa* only colonizing the apical surface of the midgut lumen, beginning from a dorsal view and ending in a ventral view of the midgut epithelium.
(AVI)
