# Supplementary figures and images for: Bacterial Infection and Immune Responses in Lutzomyia longipalpis Sand Fly Larvae Midgut
Source: PLoS Negl Trop Dis. 2015 Jul 8;9(7):e0003923. doi: 10.1371/journal.pntd.0003923 (PMC4495979; doi:10.1371/journal.pntd.0003923)

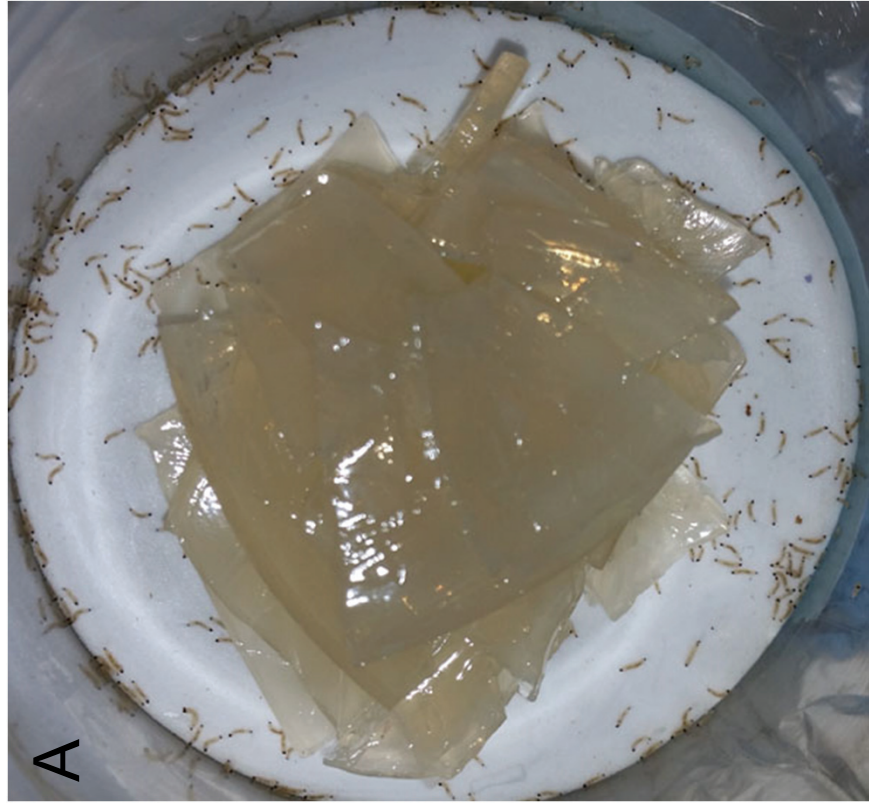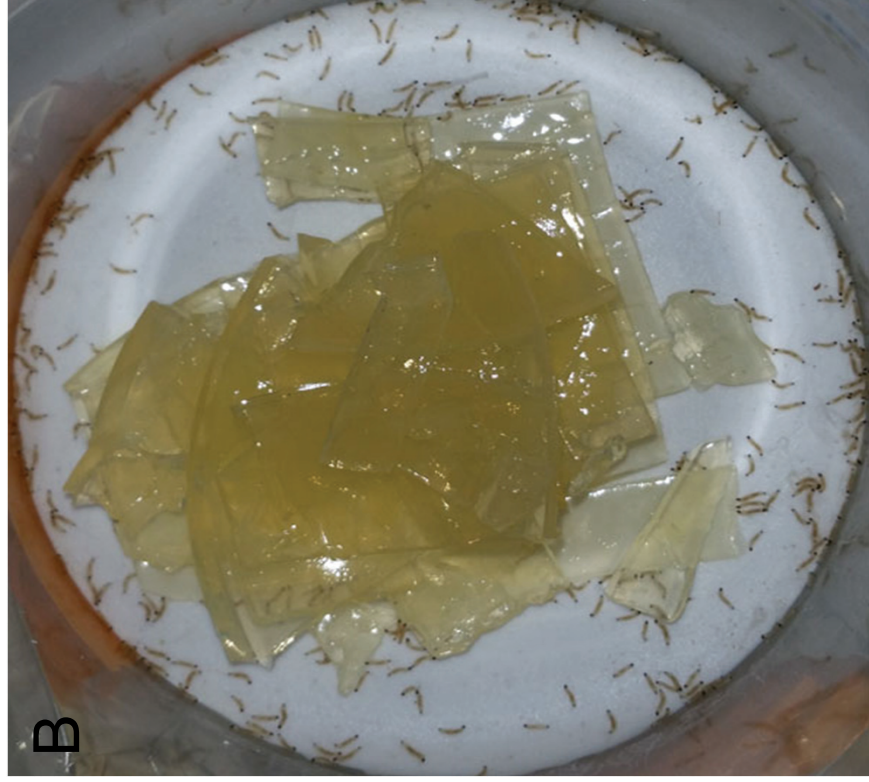

Supplement: S1 Fig — After overnight incubation on LB-agar plus antibiotics, each bacterial lawn was cut and fed to 3rd instar larvae. On the left (A) the larvae are feeding on EGFP-expressing Bs, while on the right (B) the larvae are feeding on GFP-expressing Pa. (PDF) [file pntd.0003923.s001.pdf]

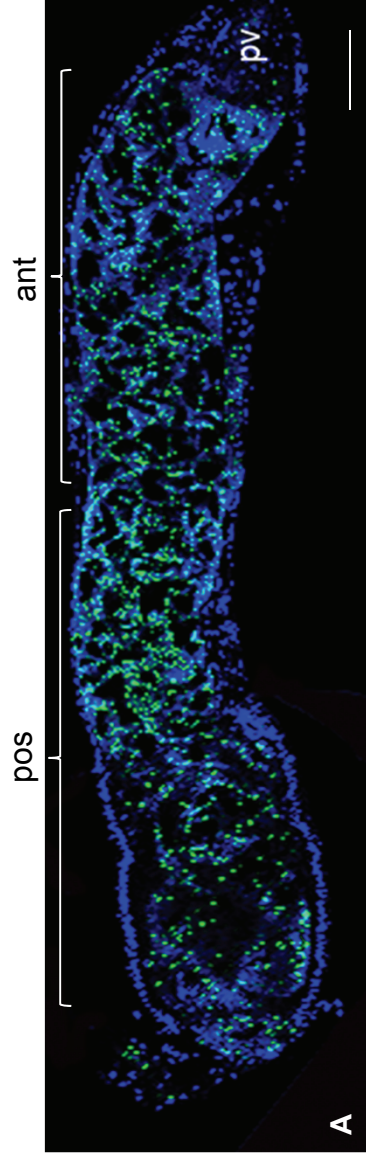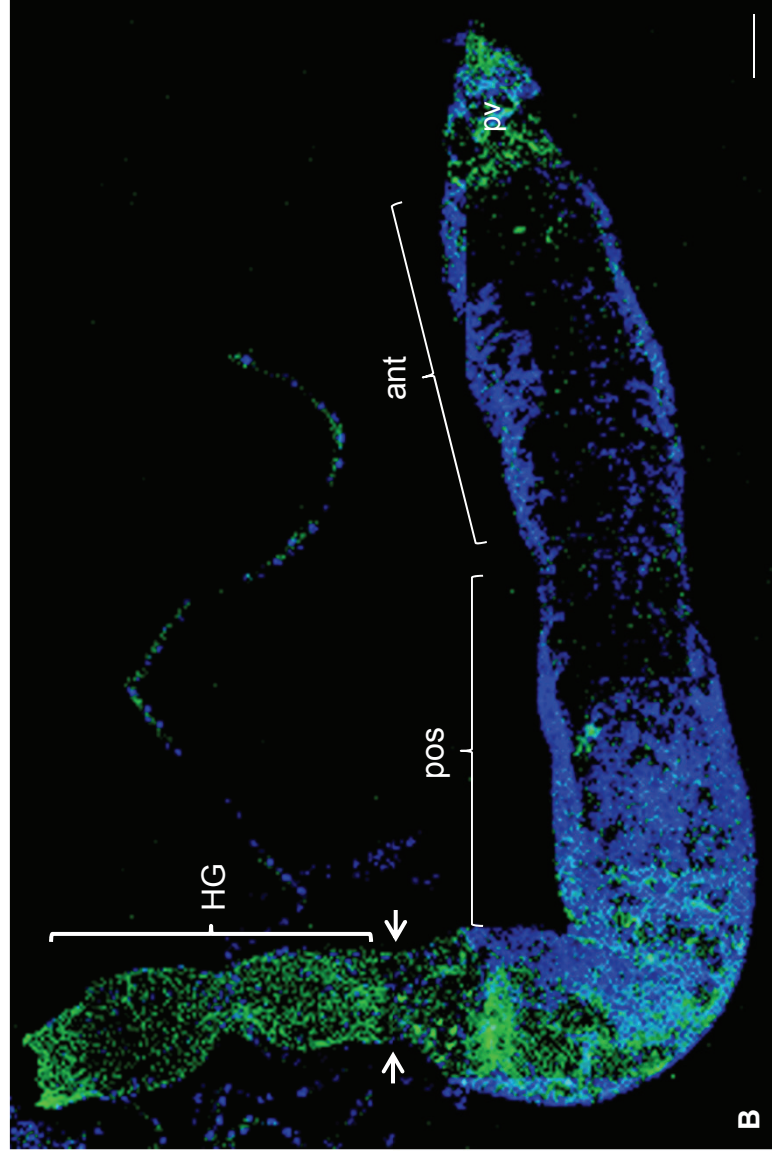

Supplement: S2 Fig — Larval guts were imaged using a resolution of 512 x 512 (number of pixels per tile). Ingested food is moved from right (proventriculus–pv) to left, towards to posterior midgut and hindgut. EGFP-expressing Bs-infected (A) and GFP-expressing Pa-infected (B) midguts are shown. Posterior (pos) and anterior (ant) midgut are marked. Arrowheads indicate the separation between midgut and hindgut. Bars = 100 µm. (PDF) [file pntd.0003923.s002.pdf]

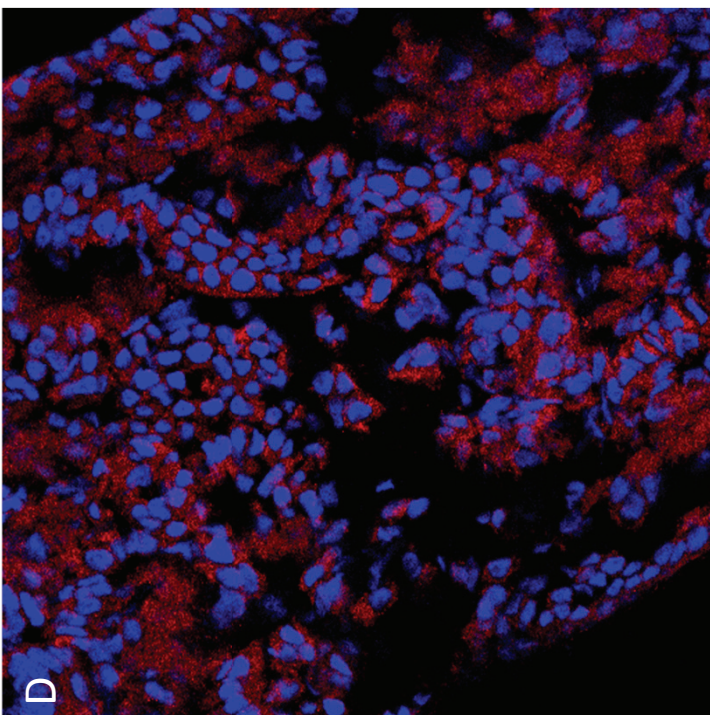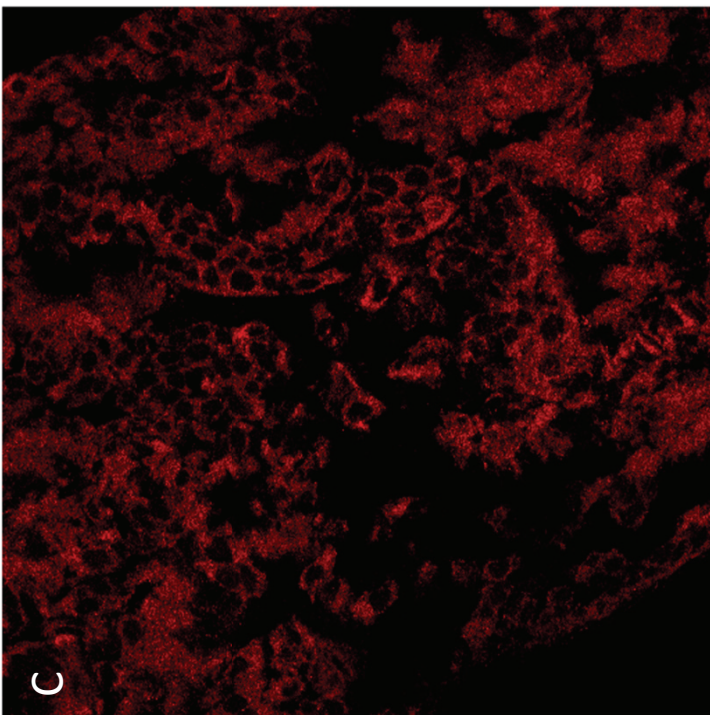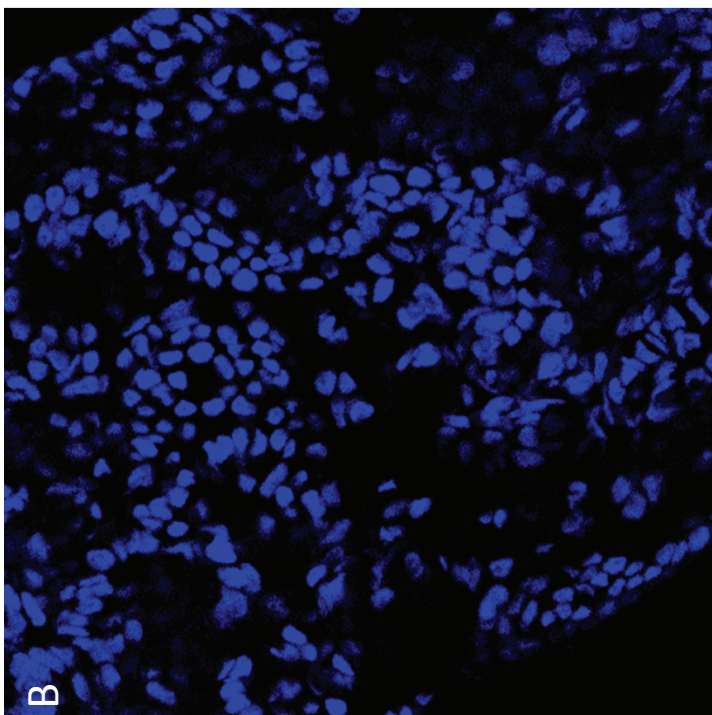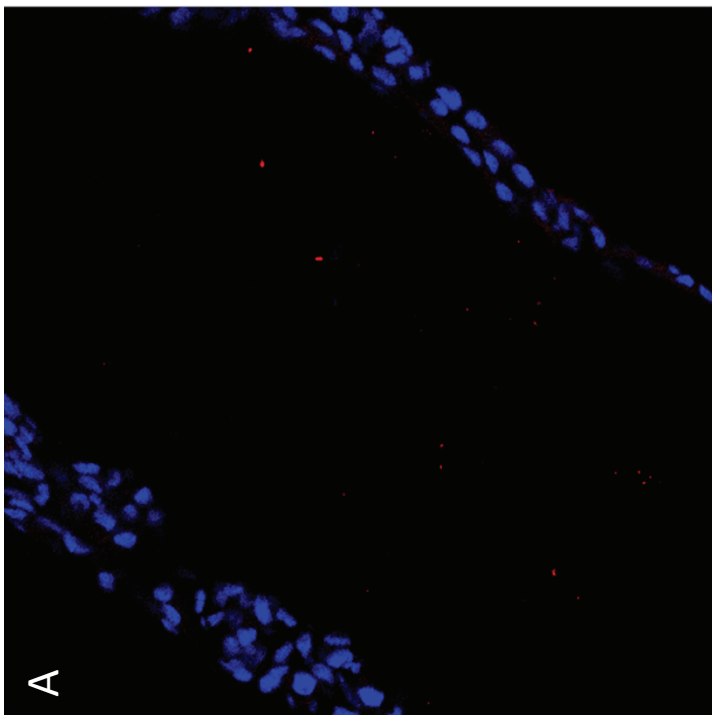

Supplement: S3 Fig — Ingestion of Paraquat induces a detectable and systemic apoptotic response in the cytoplasm of midgut epithelial cells 12h post feeding. A) Merge of caspase3 and DAPI stained nuclei for larvae fed only LB-agar medium in the anterior midgut versus B) larvae with LB-agar supplemented with Paraquat visualized with DAPI 12h post infection (anterior midgut). C) Immuno-staining for cleaved caspase3 in Paraquat fed larvae. D) Merge of B and C. Bars = 50 µm. (PDF) [file pntd.0003923.s003.pdf]

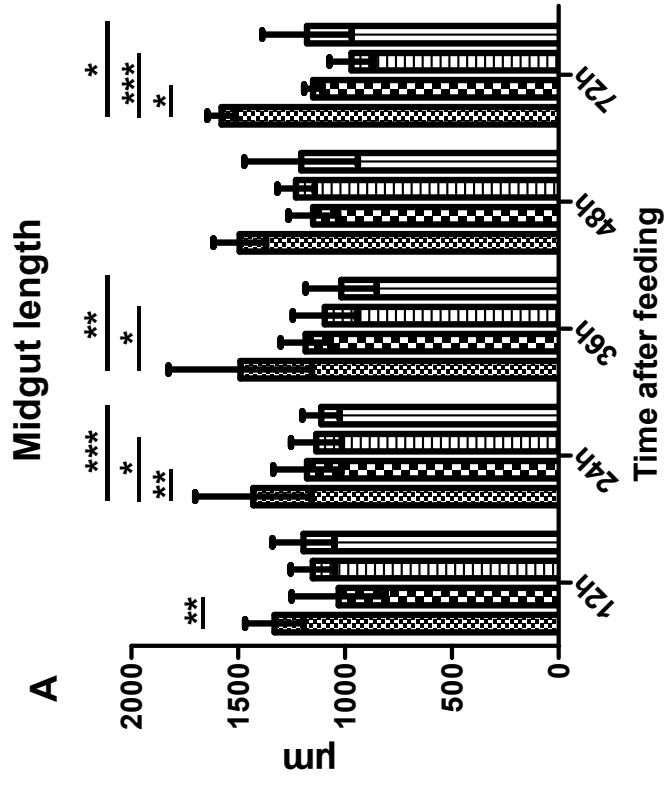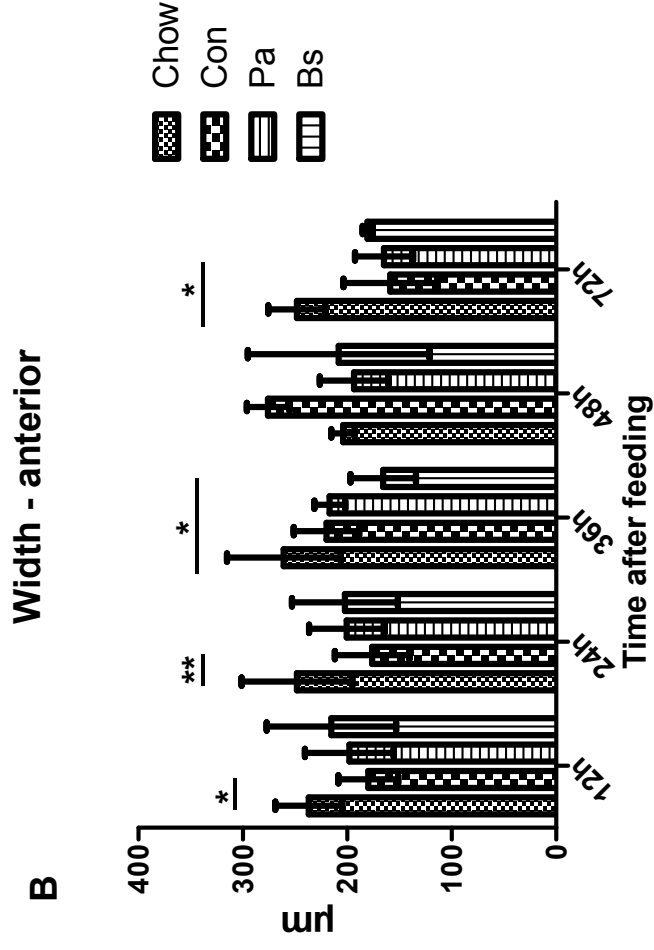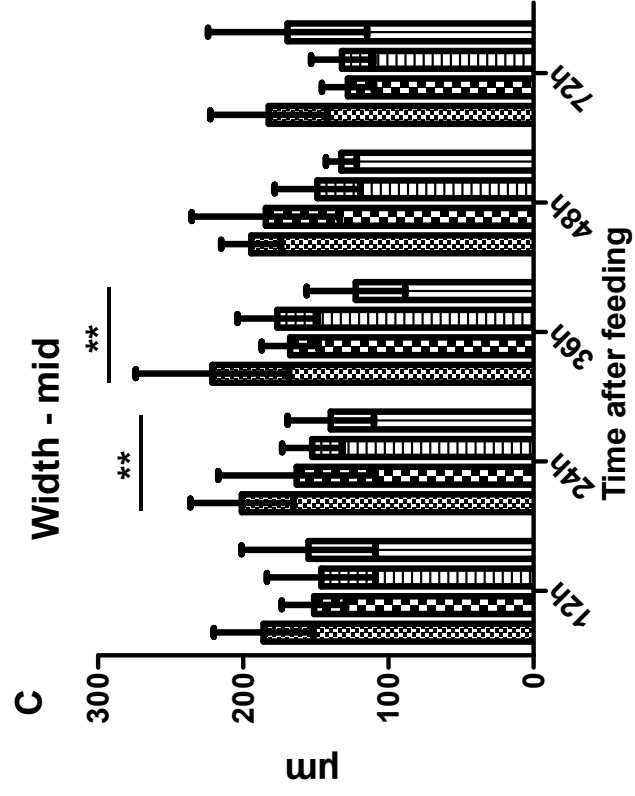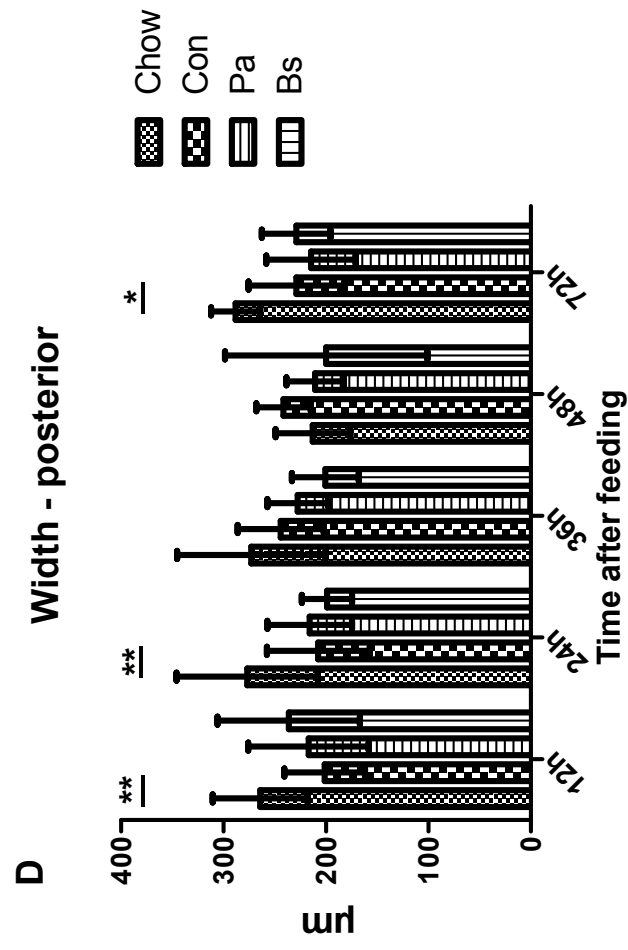

Supplement: S4 Fig — L. longipalpis 3rd instar larvae were fed on either regular sand fly larval food (50% rabbit feces + 50% rabbit food), or on LB-agar with or without bacteria, for up to 72 hours. Larval midguts were dissected and measured for length, and width in the anterior, middle, and posterior regions of the gut. Panels: A, midgut length; B, width of the anterior portion of the midgut; C, width of the middle portion of the midgut; D, width of the posterior midgut. (PDF) [file pntd.0003923.s004.pdf]
