# Supplementary material for: Bacterial Infection and Immune Responses in Lutzomyia longipalpis Sand Fly Larvae Midgut
Source: PLoS Negl Trop Dis. 2015 Jul 8;9(7):e0003923. doi: 10.1371/journal.pntd.0003923 (PMC4495979; doi:10.1371/journal.pntd.0003923)
Supplement: S1 Table — (DOCX) [file pntd.0003923.s005.docx]

| **Gene Name** | **Symbol** | **Sequence** |
| --- | --- | --- |
| Dual Oxidase F | DUOX | 5'-GGCAAGACGGAAGACAAG-3' |
| Dual Oxidase R | DUOX | 5'-TCAACAAGGGAACGACATC-3' |
| Immuno-Modulatory Peroxidase F | IMPer | 5'-CTGTGTGCGTGATAAATGTC-3' |
| Immuno-Modulatory Peroxidase R | IMPer | 5'-GTGGTAGGTGCTGGTGAAG-3' |
| Immunodeficiency F | IMD | 5'-GGTGAACAACACTCAAGCAT-3' |
| Immunodeficiency R | IMD | 5'-GTTACTCTGGGTCTGGGAGA-3' |
| Domeless F | Dome | 5'-TCAAACACACCCCAAAATAC-3' |
| Domeless R | Dome | 5'-ACGCCTCTCAATCACCATA-3' |
| AttacinA F | AttA | 5'-ATGGAATGACCTCTGTGGAT-3' |
| AttacinA R | AttA | 5'-AGCGATGAGAAAGACCAAGT-3' |
| Pirk F | Pirk | 5'-AAGATGAGTGGGAGTGAGAAG-3' |
| Pirk R | Pirk | 5'-CCAACAATACGCAAATGG-3' |
| USP36 F | USP36 | 5'-CTACGAACTGGAAGATGGTG-3' |
| USP36 R | USP36 | 5'-GATTTTGTCTCTGGCTGATG-3' |
| Vein F | Vein | 5'-CGCAATGGATGAGAACAC-3' |
| Vein R | Vein | 5'-TGAGCAATACCTACGCTGAC-3' |
| Defensin1 F | Def1 | 5'-GCCTGTGTGTTGTAGTTCT-3' |
| Defensin1 R | Def1 | 5'-GCATCTCCCCATCCTGTT-3' |
| Ribosomal Protein s6 | LlS6 | 5'-TCCCCTGGGTGATGAGTGG-3' |
| Ribosomal Protein s6 | LlS6 | 5'-CCTTTGTGCGTGGCTTCTTCC-3' |

**Supporting Table S1 – Primer list**

Primers were based on sequence information obtained from various databases. F and R in each primer indicate the forward and reverse primers used, respectively.
